# Vulnerability and Resilience in Patients with Chronic Pain in Occupational Healthcare: A Pilot Study with a Patient-Centered Approach

**DOI:** 10.1155/2018/9451313

**Published:** 2018-12-02

**Authors:** Birgitta Peilot, Paulin Andréll, Johan Gottfries, Annelie J. Sundler, Clas Mannheimer

**Affiliations:** ^1^Department of Molecular and Clinical Medicine/Pain Centre, Institute of Medicine, Sahlgrenska Academy at the University of Gothenburg, Sahlgrenska University Hospital, Gothenburg, Sweden; ^2^Department of Chemistry and Molecular Biology, Gothenburg University, Sweden; ^3^Faculty of Caring Science, Work Life and Social Wellfare, University of Borås, Sweden

## Abstract

**Objectives:**

The aim of this pilot study was to describe vulnerability and resilience and possible subgroups in patients with chronic work related musculoskeletal pain in occupational healthcare. A second aim was to evaluate a patient-centered approach.

**Methods:**

This study was based on consecutive patients with chronic pain, seen by the same physician and sick-listed full or part time three months or longer. They were included during a period of three months. Patient reported outcome measures (PROM) were administered at baseline and at follow-up after 8 months. A patient-centered approach was applied where the patient's whole situation was taken into account.

**Results:**

A dominance of an insecure dismissing attachment pattern and a subnormal sense of coherence (SOC) was reported both at baseline and at follow-up. The patients (n=38) reported significant improvement of pain severity (*p*=0.01), pain interference (*p*=0.001), life control (*p*=0.01), affective distress (*p*=0.02), and dysfunction (*p*=0.001) on the multidimensional pain inventory (MPI) and fewer patients were sick-listed full time at follow-up (13 patients versus 21). By means of multivariate data analyses this change in MPI was confirmed and was also correlated with a significant increase in health related quality of life (HRQoL). Moreover subgroups with different outcome at follow-up were identified according to attachment pattern and subgroups on MPI.

**Conclusion:**

A patient-centered approach may be of value for patients with chronic pain in occupational healthcare, improving pain and dysfunction. Patients with chronic pain are a heterogeneous group where outcome of treatment might be influenced by individual resilience and/or vulnerability.

## 1. Introduction

Chronic pain is defined as pain lasting for more than three months [1]. In a survey of chronic pain in Europe, 19% of the respondents willing to participate had moderate to severe chronic pain on a Numeric Rating Scale [2]. Pain that persists for months and years will affect all aspects of a person's life, physical, emotional, interpersonal, and social. This biopsychosocial model is the basis of a patient-centered approach [3]and also of a cognitive-behavioral treatment for chronic pain [4, 5]. Most people with acute pain will recover and all patients with chronic pain do not become physically and emotionally disabled [4]. There are few clear-cut answers to what factors are the most important in the development of chronic pain. Patients with chronic pain are not a homogenous group and previous research has recommended both a medical and psychosocial approach [3, 6]. A stress-diathesis perspective of the development of chronic pain has been proposed, where the stress of living with chronic pain activates individual vulnerability or resilience factors [7, 8] Hereby vulnerability in terms of an insecure attachment pattern or a weak sense of coherence (SOC) might influence the development of depression, anxiety, and dysfunctional coping strategies [8–10]. According to Bowlby's theory of attachment [11], early attachment pattern between the child and its mother also plays an important role in adult life where pain and illness may activate attachment behaviors, meant to preserve the survival of the organism. Bowlby's attachment theory may allow for identification of subgroups of patients with chronic pain [12, 13]. The SOC scale was developed by Antonovsky [14] and measures the individual's capacity to respond to stressors by flexible and appropriate coping strategies. A secure attachment pattern and a strong SOC are possible factors of resilience. Resilience is defined as a person's ability to adapt to and manage stress and harm [15]. In contrast to a view of disease focusing on pathology the salutogenic model developed by Antonovsky focuses on factors that support human health and well-being [16, 17]. Turk and Okifuji [4] have suggested that chronic pain should be seen as a chronic disease just like other chronic diseases where expectations of cure are limited. These patients need follow-up with a focus on maintenance and enhancement strategies where the goal is recovery from the consequences of pain and disability. Recovery is a process of change of one's attitude, values, and goals. It is a way of living a satisfying and meaningful life even with the limitations caused by illness [3]. A patient-centered approach where the patient is empowered to be an active subject in creating a new meaning in her/his life, as described by Malterud and Hunskaar, can hereby enhance recovery [3]. Early interventions within 1-3 months in patients with pain are preferred to prevent later development of a more complex chronic pain according to the official final report of the Rehabilitation Council in Sweden 2011 [18]. The importance of the role of occupational healthcare in the contact between the patient and the work place and for early rehabilitation was also emphasized. There are few studies, to our knowledge, regarding patient-centered approaches of chronic pain in occupational healthcare [19]. The aim of this pilot study was to describe vulnerability, resilience, and possible subgroups in patients with chronic work related musculoskeletal pain in occupational healthcare. A second aim was to evaluate a patient-centered approach.

## 2. Materials and Methods

During a period of three months all consecutive patients sick-listed for ≥3 months due to chronic musculoskeletal pain were included. Exclusion criteria were insufficient knowledge of the Swedish language and not being able to independently complete the questionnaires. A biopsychosocial approach was used and the patient's whole situation was taken into account in the treatment with a focus on empowerment as described by Malterud and Hunskaar [3] and in a review of effective physician-patient communication [20]. The patients were seen regularly, on average once a month, by the physician. Hereby the tasks of the clinician in the encounters were to pursue the medical agenda as well as the patient's agenda [3, 21]. It also implied a cognitive approach with collaboration between the patient and the physician on equal terms [22]. The physician (BP) was specialized in rehabilitation medicine and was also a certified cognitive psychotherapist. The pharmacological treatment of patients in this study was mainly with nonopioid substances such as Paracetamol, NSAID, and also low dose Amitriptyline. Some patients with depression and sleep disturbances were treated with antidepressants and Zopiclone or Zolpidem. A physiotherapist and a nurse were available for conventional physical training, to give ergonomic advice and for contacts with the employer.

### 2.1. Patient Reported Outcome Measures

The questionnaires were administered in the clinical setting as a rule at baseline and after 8 months of treatment. However, at follow-up the questionnaires were mailed to a few patients (after a telephone contact).

#### 2.1.1. Relationship Scales Questionnaire

The Relationship Scales Questionnaire (RSQ) is a self-assessment instrument for the assessment of attachment patterns according to Bowlby's Attachment theory developed by Griffin and Bartholomew [23]. A four-category model is obtained from either a positive or negative view of self and others resulting in one secure and three insecure attachment patterns: dismissing, fearful, and preoccupied. The RSQ consists of 17 statements where participants rate each statement on a seven point scale ranging from 1 to 7. Scores are then averaged across the five statements for secure, five for dismissive, four for fearful, and four for preoccupied attachment styles. The RSQ yields scores of all subscales of attachment. Participants can then be described as having varying degrees of each attachment style. The subscales have shown moderate consistency with Cronbach's alpha coefficients for scores ranging from 0.45 for preoccupied and 0.65 for secure to 0.7 for dismissing and 0.76 for fearful [24]. The RSQ has also shown convergent and discriminant validity for these dimensions and a high test-retest reliability over an 8-month period [23].

#### 2.1.2. Sense of Coherence

The SOC used in this study contains 29 items. The concept sense of coherence was developed by Antonovsky to describe health promoting resources based on three components of this concept: comprehensibility, manageability, and meaningfulness [14]. Higher scores indicate a better value of SOC [14]. The internal consistency for SOC is good, with a Cronbach's alpha ranging from 0,82 to 0.95 [25]. The test-retest correlations have shown a considerable stability, 0.54 over a two-year period.

#### 2.1.3. Multidimensional Pain Inventory

The MPI is a self-assessment instrument measuring psychosocial, cognitive, and behavioural effects of chronic pain developed by Turk and Rudy [26, 27]. The MPI (version 2) used in this study is a 61-item questionnaire. The answers of the questionnaire are grouped into 13 scales. A subgroup classification on the basis of significant differences of responses to 9 of the scales of the MPI (pain severity, pain interference, affective distress, support, distracting responses, solicitous responses, punishing responses, general activity, and life control) is possible to obtain by means of cluster analysis in a specific computer program [28]. These profiles or subgroups are labelled: adaptive coper (AC), anomalous (ANOM), interpersonally distressed (ID), hybrid, and dysfunctional (Dys). The raw scores are hereby transformed into a 0-100 scale. The subgroups are characterized by different levels of pain intensity and pain interference, affective distress, and reactions of significant others. AC patients report less pain severity and pain interference, lower levels of affective distress, and higher activity levels compared with patients in the ID, hybrid, and Dys subgroups. The MPI was based on a normative sample of chronic pain patients and has a good internal consistency [27].

#### 2.1.4. Beck Anxiety Inventory

The BAI measures the severity of anxiety during the past week with a 21-item self-report inventory using a four-point scale ranging from 0 (no symptoms present) to 3 (severe symptoms). The standard cut-offs are as follows: 0-7 indicates minimal anxiety, 8-15 indicates mild anxiety, 16-25 indicates moderate anxiety, and 26-63 indicates severe anxiety [29]. It has shown a high internal consistency of 0.90-0.92 and the test-retest reliability is satisfactory (0.75) [30]. Convergent validity with other self-report anxiety scales has been estimated (0.35-0.69).

#### 2.1.5. Beck Depression Inventory

The BDI is a widely used questionnaire for assessing the severity of depression and is sensitive to changes over time [31]. The standard cut-offs are as follows: 0-9 indicates minimal depression, 10-18 indicates mild depression, 19-29 indicates moderate depression, and 30-63 indicates severe depression. Test-retest correlations vary between 0.48 and 0.86 in accordance with the natural development of depressive symptoms [32]. In chronic pain high correlations (0.73) have been reported with clinical ratings of depression indicating good construct validity [32, 33]. In this study the BDI-1A was used.

#### 2.1.6. Pines' Burnout Measure

The Pines' Burnout Measure assesses three components of burnout: physical exhaustion, mental exhaustion, and emotional exhaustion. It consists of a 21-item scale. Of the 21 items, 17 are negative and four are positive. All items are responded to on a 7-point scale ranging from1 to 7. The score is determined as the mean response to all items with positive items reversed. The cut-off value for burnout is >4.0 [34, 35]. The Pines' Burnout Measure has been shown to have high validity and reliability [34].

#### 2.1.7. Short Form 36

The Short Form 36 (SF-36) evaluates physical and psychological aspects of health related quality of life (HRQoL). The items are grouped into eight subscale scores: physical functioning (PF), role limitations caused by physical problems (RP), bodily pain (BP), general health (GH), energy/vitality (VT), social functioning (SF), role limitations caused by emotional problems (RE), and mental health (MH). The subscales range from 0 to 100, where a higher value indicates a better HRQoL [36]. The SF-36 has been translated and adapted to Sweden and has high validity and reliability [37–39].

### 2.2. Statistical Methods

For comparison within the group over time the Wilcoxon signed rank test was used in SPSS 12.0.1. All statistical tests were two-tailed and conducted at a 5% significance level.

In a next step Multivariate data analyses (MVDA) using Principal Component Analysis (PCA) [40] and Two-Way Orthogonal Partial Least Squares Analysis (O2PLS) [41, 42] were performed as an explorative method in order to confirm the results and to discover possible correlations and patterns of variables in the patient group. MVDA is a factor method used to uncover the latent structures or dimensions when a set of relevant data and variables are analyzed together, for interpretation based on the yielded correlation, translated to variables and reducing “noise”, i.e., uncorrelated data (further description of MVDA in supplemental information).

## 3. Results 

### 3.1. Characteristics of Patients

**Figure 1:**
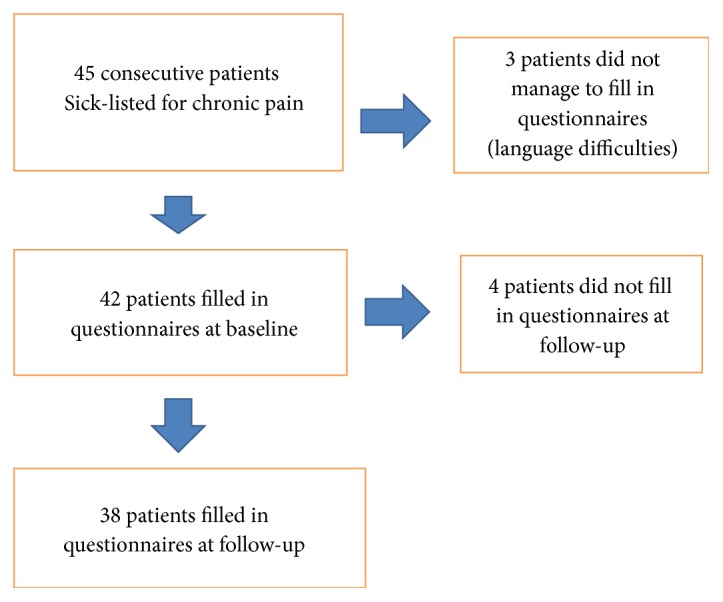
Flow chart for 45 consecutive patients with chronic pain in occupational healthcare sick-listed three months or longer.

During the inclusion period 45 patients were identified, and, of those, 35 women and 7 men agreed to participate in the study. Three patients were not eligible due to insufficient Swedish language competency, hence not being able to independently complete the questionnaires. In the remaining group (n=42) (Figure 1), 50% were on full-time sick leave and 50% on part-time sick leave. The pain diagnoses were fibromyalgia (36%), chronic regional pain (33.%), chronic widespread pain (19%), lumbago (10%), and whiplash trauma (2.%). Local pain diagnoses were mainly myofascial and/or degenerative cervicobrachial, work related pain disorders. No specific neurogenic pain syndromes were diagnosed. The duration of the sick leave was on average 18.5 months, not including two of the patients who had a temporary full time sick benefit. The average age was 44 years (range 25-58 years). In the results of the multivariate analyses patients above mean age are referred to as older and those below mean age are referred to as younger. At follow-up 38 patients completed the questionnaires (Figure 1). Four patients could not be reached or did not answer the questionnaires on request. Missing data for all outcome measures are provided in supplemental information Tables S1A-C. At baseline 21 patients were sick-listed 100% versus 13 patients at follow-up. No patients worked full time at baseline but two patients worked full time at follow-up. The remaining worked part time 25-75% at follow-up (Figure S 1 in supplemental information). This change was significant (*p*= 0.01*).*

#### 3.1.1. Patient Reported Outcome

As shown in Table 1, a dismissing attachment was the dominant pattern at baseline and at follow-up. However, there was a significant increase in the secure pattern at follow-up (*p*=0.01) (Table 1). SOC at baseline was reported with a mean value of 125 and at follow-up 128 (*p*=0.35), indicating a minimal impairment of the mean value of SOC both at baseline and at follow-up (Table 1). At baseline the mean value for anxiety, assessed with BAI, was 16.4 and for depression, assessed with BDI, was 18.6. This implies a moderate level of anxiety and a mild to moderate level of depression (Table 1). No significant changes were seen at follow-up. The distribution of patients according to minimal-mild, moderate, and severe anxiety and depression at baseline and follow-up is shown in a histogram (Figures S 2A-B). The patients also scored below the cut-off value for burnout, 4.0, both at baseline and at follow-up with 3.7 versus 3.6. The reported values of MPI at follow-up showed a significant improvement of pain severity (*p*=0.01), pain interference (*p*=0.001), life control (*p*=0.01), affective distress (*p*=0.02), and dysfunction (*p*= 0.001) (Table 2). Subgroups in MPI were also assessed, and there was a dominance of Dys and ID subgroups at baseline and at follow-up, yet with an increase of patients in the AC subgroup at follow-up from 4 to 8 patients (Figure S 3). In SF-36 there was no significant change at follow-up (Table S 2).

#### 3.1.2. Correlations and Subgroups Assessed with PCA and O2PLS

**Figure 2:**
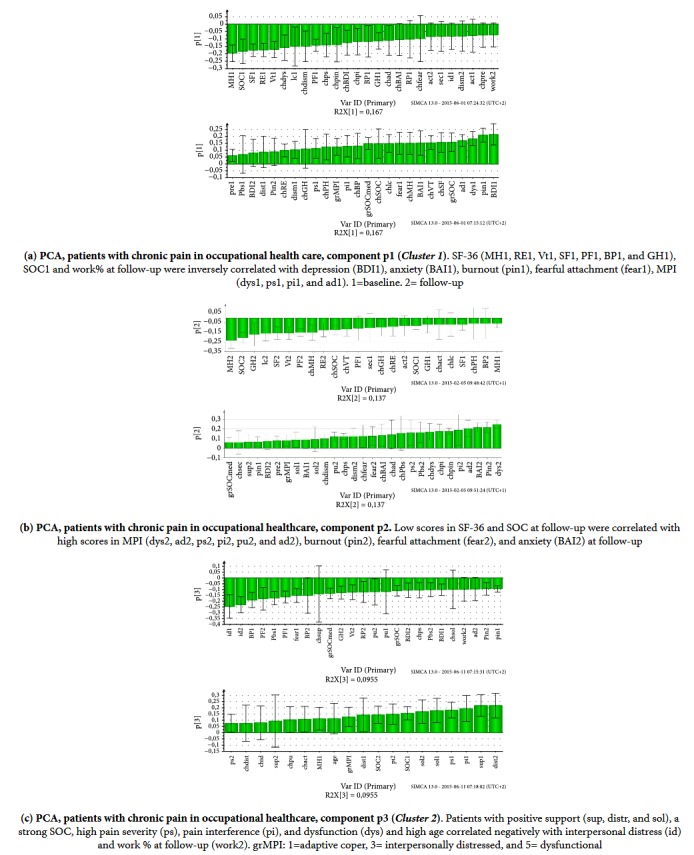


In the PCA three significant principal components (p1-3) explained 40% of the variation of the data, (R^2^X=0.40) with a cross-validated prediction, Q^2^X=0.17 (Figure S 4). Component p1 and p3 modeled items in MPI, RSQ, SOC, BAI, BDI, burnout, and HRQoL (Figures 2(a) and 2(c)) and component p2 modeled change variables (Figure 2(b)). In the loading histogram of the first principal component HRQoL and work at follow-up were negatively correlated with anxiety, depression, and pain associated symptoms.* Cluster 1* in p1 had a resemblance of two subgroups in MPI (AC and Dys). AC correlated inversely with the loadings of a Dys subgroup (Figure 2(a)). In the loading histogram of the third principal component p3 (Figure 2(c)) a second cluster (*Cluster 2*) with another Dys subgroup was distinguished inversely correlated with the loadings of an ID subgroup. The patients in this Dys subgroup were older. They had good support from significant others and reported a higher HRQoL and a strong SOC but also had a positive correlation with dysfunction and pain in MPI.

Patterns and clusters of subgroups can be seen in loading and score plots combining two principal components. Clusters 1 and 2 are identified in Figures S 5-6.

**Figure 3:**
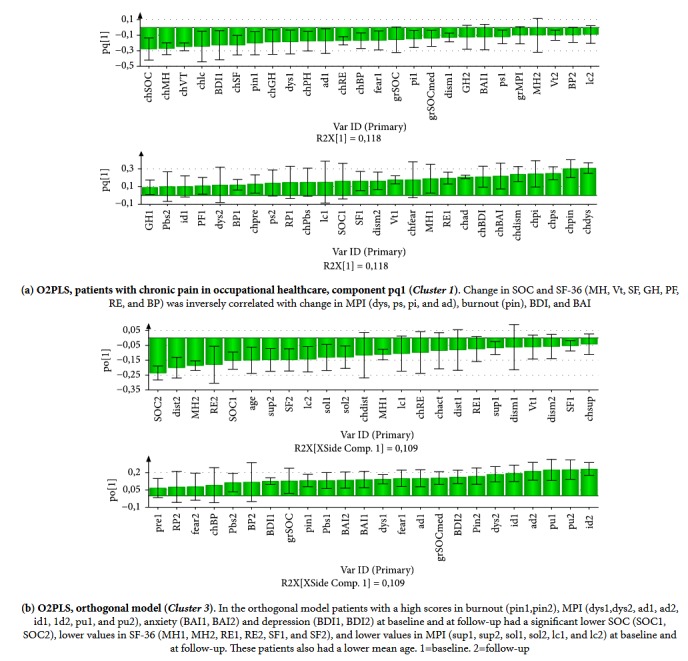


To enhance the correlation pattern provided by PCA, change variables were chosen from relevant variables in the previous PCA with reasonable large confidence intervals to be used as Y variables in an O2PLS analysis. In total 13 change variables were selected as Y variables whereas all other variables were kept as X variable. The rendered model comprised three predictive components (pq1-3) and one orthogonal component (po1). The explained variance R^2^Y was 0.54 and the prediction Q^2^ Y was estimated to 0.34 (Fig.  S 7A). The change variables had a reasonably high explained variance and prediction using the present study material, with the lowest prediction of the variable for work at follow-up (Figure S 7B). The loading histogram of the first predictive component pq1 is shown in Figure 3(a). In this model, patients with a negative change in dysfunction, affective distress, and pain correlated with a significant positive change in HRQoL. This cluster also corresponds to* Cluster 1* in the previous PCA.

Significant variations in the orthogonal component po1 (Figure 3(b)) comprised structured data that do not correlate with the Y matrix structure which was obtained in the first change model, i.e., no correlation with any of the O2PLS nominated Y-vectors. The orthogonal component modeled a cluster of patients (*Cluster 3*) with high levels of dysfunction, affective distress, and interpersonal distress in MPI, anxiety, depression, burnout, and fearful attachment at baseline and follow-up. These patients also had a significant lower SOC and mental health, physical function, social function, and vitality in SF-36 at baseline and follow-up and a lower mean age. This model also implied an opposite interpretation where a subgroup of older patients with higher scores of items in SF-36, SOC, and support from significant others at baseline and follow-up also had lower scores of pain severity and dysfunction in MPI and lower scores of burnout and depression at baseline and follow-up. Thus this orthogonal model represents a third cluster of patients comprising adaptive and a mixture of very dysfunctional or interpersonally distressed patients, all with a low potential of change.

**Figure 4:**
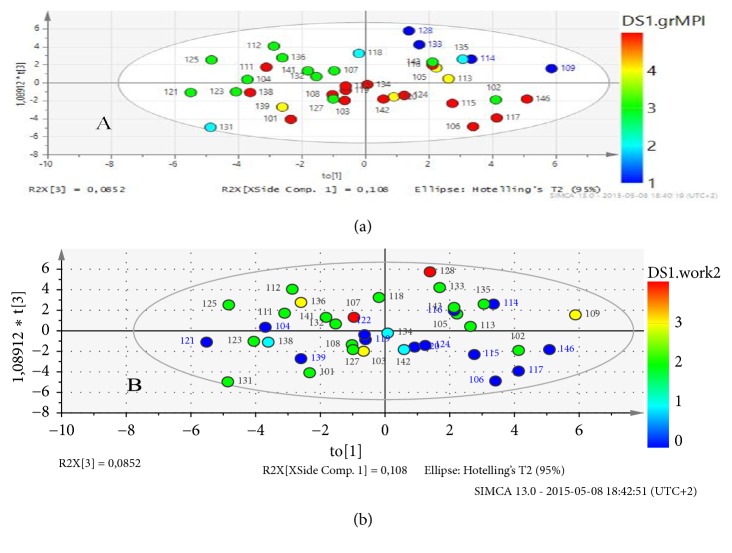
**O2PLS, patients with chronic pain in occupational healthcare. Score plots (to/t3). Score plot (a) patient scores colored according to subgroups in MPI** (1= adaptive coper, 2= anomalous, 3= interpersonally distressed, 4= hybrid, and 5= dysfunctional).** Score plot (b) patient scores colored according to work at follow-up**. (0=no work, 1=25%, 2=50%, 3=75%, and 4=100%). Patients colored red (dysfunctional) in plot (a) mainly were colored blue (low work %) in plot (b).

The third predictive component in the O2PLS pq3 (Figure S 7C) modeled variables correlated with work at follow-up. According to loadings pq3 _corr_ in the third predictive component of the O2PLS, working percent at follow-up had a correlation of 0.43 with high scores in SF-36 and interpersonal distress and low scores in MPI for pain and dysfunction both at baseline and at follow-up (Figure S 7D). This cluster had a resemblance with Cluster 2 identified in the previous PCA and corresponded mainly to the subgroups AC and ID on the MPI. In a loading plot (Figure 4(a)) Cluster 2 in component pq3 was combined with the orthogonal component po1 (Cluster 3). In the corresponding score plots (Figure 4) work at follow-up was associated with patients belonging to the AC and ID subgroups in MPI. Hence, those patients who were working at follow-up were less dysfunctional.

## 4. Discussion

Patients in the present study, sick-listed for chronic pain in occupational healthcare, had a dominance of dismissing, insecure attachment pattern and also a subnormal SOC. Moreover the proportion of patients belonging to dysfunctional and interpersonally distressed subgroups in MPI was larger than expected, indicating a possible vulnerability [26, 43]. A patient-centered approach may be of value for patients with chronic pain in occupational healthcare improving pain and dysfunction according to MPI (Table 2). Both very low and high values of anxiety, depression, SOC, and HRQoL were reported both at baseline and at follow-up indicating heterogeneity of the group which was also shown by multivariate data analyses with PCA and O2PLS. This heterogeneity was in line with previous research [6]. A patient-centered approach may support a more individualized treatment adapted to the patient's special needs. There are few randomized studies of patient-centered care in chronic pain mainly because of the individual nature of patient-centered care and problems with objective assessment [19, 44].

**Figure 5:**
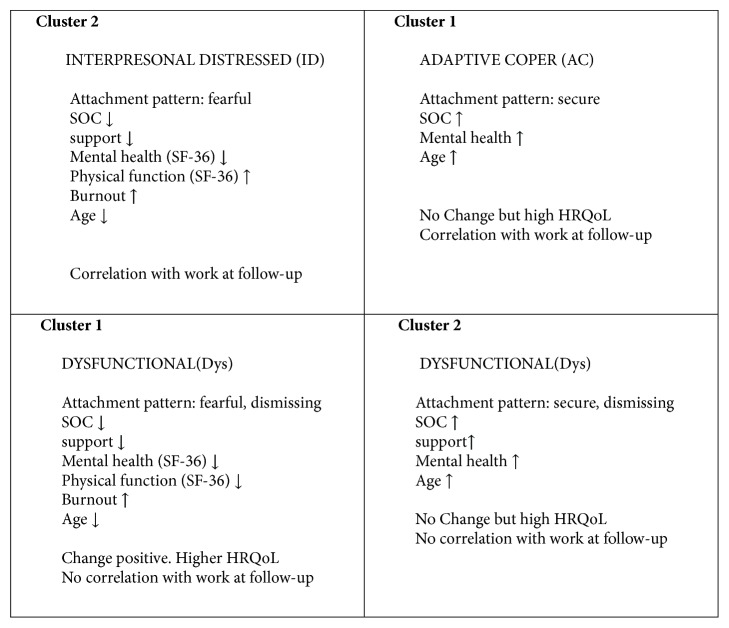
**Correlations and clusters of observations in patients with chronic pain in occupational healthcare**. Two clusters were identified with PCA and O2PLS** corresponding to subgroups in MPI:** Cluster 1 with AC inversely correlated with Dys (low support) and Cluster 2 with ID inversely correlated with DYS (high support).

A subgroup classification on the basis of significant differences in responses to 9 of the scales of the MPI is possible to obtain by means of cluster analysis in a specific computer program developed by Turk and Rudy [28]. In the present study similar subgroups have been identified both with this specific computer program and in the multivariate data analyses by PCA/O2PLS, summarized in Figure 5. The clusters in the present study had similarities with the subgroups obtained in another study by Rovner et al. [45], where pain acceptance and active engagement assessed on the Chronic Pain Acceptance Questionnaire (CPAQ-8) correlated with HRQoL and positive effects of rehabilitation.

In a summary of the validity of subgrouping in MPI [6] Turk has concluded that the psychosocial dimension of chronic pain may be independent of physical pathology, which was in line with the findings in the present study where patients with higher values of pain and dysfunction in* Cluster 2* also correlated with higher values of HRQoL and SOC. The subgrouping in MPI also has been useful for treatment matching where patients in the Dys and ID subgroups seem to have other needs of treatment than patients in the AC subgroup. Patients in the ID subgroup may have problems with interpersonal skills and many patients in this subgroup make very little progress in conventional rehabilitation [6]. Patients in the Dys subgroup on the other hand often have higher levels of emotional distress, a feeling of low control, and high pain interference. They often show significant improvement with support and focus on cognitive factors [6]. The patients in the AC subgroup often do not change or even regress probably due to a floor effect [6], which was in line with the results of the present study. However, here both AC and ID subgroups correlated with a higher amount of work at follow-up (Figure 4(c)). Patients with more support tended to be older, to have higher values of SOC and HRQoL but had a lower potential for change and work at follow-up. The patients in the mixed Dys and ID subgroup in* Cluster 3* were those who had signs of the highest vulnerability and a low support. These mainly younger patients might have needed more therapeutic support, while older patients with good support from significant others and higher values of HRQoL might need fewer interventions [46]. The findings in the present pilot study need to be confirmed in further randomized studies and if possible with specific treatment matching in the different subgroups. Hereby it would also be interesting to use the QPAQ-8 questionnaire as described by Rovner et. al. [45] in combination with SOC, MPI and HRQoL.

### 4.1. Clinical Implications

A patient-centered approach in combination with subgrouping in MPI and assessment of SOC, anxiety, depression burnout, and HRQoL may give important knowledge and understanding of individual resilience and vulnerability among patients with chronic pain. The factor structure and psychometric properties of the MPI have been replicated in numerous studies in several countries to assess chronic pain from a cognitive behavioural perspective [47]. Hereby the treatment can be adjusted to individual needs. Among patients with chronic pain in primary healthcare a strong SOC has been associated with a higher HRQoL and self-efficacy [48]. While patients with a strong SOC and adaptive coping strategies can manage with less support, patients with dysfunctional strategies and more affective symptoms may have a good outcome with more support and a focus on cognitive factors. However, not all patients will improve and it is important to recognize those patients. The study highlights the importance of identifying patients with high levels of pain, anxiety, depression, burnout, and a very low HRQoL and SOC who might need special interventions. These patients might benefit from more individualized treatment while others with good support from significant others could manage without other interventions [46].

### 4.2. Strengths and Limitations

The current pilot study was based on patients assessed by the same physician at an occupational healthcare center in a small town with 20 000 inhabitants in the western part of Sweden. In 2007 when this study was performed all patients with work related symptoms were attending the occupational health care. Ten years later many of these patients are referred to primary healthcare. During a period of three months in 2007 all consecutive patients sick-listed for ≥3 months due to chronic musculoskeletal pain were included. A longer inclusion period was not possible due to a limited time to carry out the study. All patients had the same treatment with a patient-centered approach and were seen regularly for sick-listing and follow-up during eight months. Hence this study was based on a small cohort of patients and was limited by rather few participants (n=42), especially regarding the heterogeneity of the group, short follow-up time, and the lack of a control group. In order to compensate for relatively few observations and many variables multivariate data analyses (MVDA) were performed. The multivariate projection discards noise and the latent factors (i.e., scores and loadings) become stabilized by inclusion of relevant variables. By introducing many variables at the same time multiple collinearity can be created. Most datasets in the present area provide a few underlying phenomena that describe the system. In recent years this method has been used increasingly to investigate both very small and large populations of patients with chronic pain according to systemic differences in serum and liquor metabolomics [49]. The same methods have also been used to find different patterns of biopsychosocial factors influencing vulnerability, resilience, and outcome of treatment [50, 51]. Taking into account these special features of the MVDA we argue that the statistical methods are sound and give support for our conclusions. Hereby it was possible to distinguish separate subgroups with different outcomes that might not have been recognized if only the results of the entire group had been evaluated.

The nonresponse at follow-up was 4 patients. These patients did not diverge from the group mean values at baseline but for a significant lower mean value of dysfunction and pain interference. The heterogeneity in groups of patients with chronic pain also makes it difficult to assess the effects of treatment as all patients do not benefit from the same treatment [6]. Using MVDA can hereby make it possible to distinguish different outcomes of treatment in different subgroups. A spontaneous recovery might also influence the outcome. However, meta-analyses of previous research have shown that patients on a waiting-list improve less than patients who get psychological interventions [4]. The next step is to proceed with a larger randomized study. It would also be of great interest to further investigate the possibilities of subgrouping patients with chronic pain according resilience and vulnerability, in order to individualize and thus optimize treatment.

## 5. Conclusion

A patient-centered approach may be of value for patients with chronic pain in occupational healthcare, improving pain and dysfunction. Patients with chronic pain are a heterogeneous group where outcome of treatment might be influenced by individual resilience and/or vulnerability.

## Figures and Tables

**Table 1:** RSQ (attachment pattern), SOC, BAI (anxiety), BDI (depression), and Pines' burnout measure, at baseline and at follow-up in patients with chronic pain in occupational health care.

Questionnaires	Baseline (n=42) Mean (SD) Range	Follow-up (n=32-38) Mean (SD) Range	*p* value
RSQ Attachment Fearful	3.9 (1.0)	3.6 (1.3)	0.23
	1.75-6.0	1.0-6.5	
RSQ Attachment Dismissing	4.6 (1.1)	4.9 (0.8)	0.24
	1.0-6.25	2.8-6.6	
RSQ Attachment Secure	3.8 (0.9)	4.2 (0.7)	0.01
	2.0-5.5	2.2-5.6	
Attachment Preoccupied	3.7 (0.8)	3.6 (0.9)	0.94
	1.75-5.5	1.75-6.75	
SOC	124.9 (27.7)	128.4 (26.9)	0.35
	57.0-174.0	65.0-185.0	
BAI	16.4 (10.4)	15.8 (10.0)	0.53
	2-38	2-37	
BDI	18.6 (9.3)	16.3 (9.6)	0.14
	0-49	4-44	
Pines' Burnout Measure	3.7 (1.0)	3.6 (1.0)	0.26
	1.67-6.48	1.7-5.95	

**Table 2:** MPI (Multidimensional Pain Inventory) at baseline and at follow-up in patients with chronic pain in occupational healthcare.

	Baseline (n=42) Mean (SD) Range	Follow-up (n=37) Mean (SD) Range	*p* value
Pain severity	69.3 (1.0)	63.3 (15.0)	0.01
	37.0-100.0	41.0-100.0	
Pain interference	66.4 (14.1)	61.1 (9.8)	0.001
	39.0-100.0	45.0-90.0	
Life control	42.6 (12.6)	48.4 (9.8)	0.01
	0.0-64.0	24.0-72.0	
Affective distress	58.3 (17.1)	51.0 (15.9)	0.02
	30.0-100.0	0.0-85.0	
General activity	54.4 (5.3)	54.6 (5.4)	0.38
	46.0-68.0	42.0-68.0	
Dysfunction	58.4 (10.9)	53.6 (8.3)	0.001
	26.0-86.0	40.0-82.0	
Interpersonal distress	38.3 (11.5)	38.8 (10.6)	0.96
	13.0-60.0	19.0-57.0	

## Data Availability

All data of the study is to be found in the manuscript and in supplementary material.

## Abbreviations

AC: Adaptive coper
act: General activity
ad: Affective distress
BAI: Beck anxiety inventory
BDI: Beck depression inventory
BP: Bodily pain
ch: Change
dism: Dismissing attachment
distr: Distracting responses
dys: Dysfunctional
fear: Fearful attachment
HRQoL: Health related quality of life
id: Interpersonal distress
lc: Life control
MH: Mental health
MPI: Multidimensional Pain Inventory
Pbs: Performance based scale
PF: Physical function
pi: Pain interference
pin: Pines' burnout measure
pre: Preoccupied attachment
ps: Pain severity
pu: Punishing responses
RE: Role emotional
RP: Role physical
RSQ: Relationship Scales Questionnaire
SF-36: Short Form 36
SF: Social function
sec: Secure attachment
SOC: Sense of coherence
sol: Solicitous responses
sup: Support
Vt: Vitality.

## Supplementary Materials

Supplementary Materials
* Supplemental Digital Content*.** Figure S 1. Patients in occupational healthcare**. Number of patients working 100%, 75%, 50%, and 25% at baseline (blue) and at follow-up (red). At baseline 21 patients were not working versus 13 at follow-up.** Figure S 2A**.** Patients with chronic pain in occupational healthcare**. Anxiety assessed with BAI at baseline and at follow-up. BAI<16= minimal-mild anxiety, 16-25= moderate anxiety, and >25= severe anxiety. At baseline 56% of the patients had a minimal-mild anxiety versus 59.5% at follow-up. The remaining patients reported values above that level.** Figure S 2B. Patients with chronic pain in occupational healthcare**. Depression assessed with BDI at baseline and at follow-up. BDI<19= minimal-mild depression, 19-29= moderate depression, and >29 = severe depression. At baseline 55% of the patients had a minimal to mild depression versus 69.4% at follow-up. The remaining patients reported values above that level.** Figure S 3. Patients with chronic pain in occupational healthcare. Subgroups in Multidimensional Pain Inventory (MPI) at baseline and at follow-up**. Dominant subgroups were dysfunctional (Dys) and interpersonally distressed (ID).** Figure S4. PCA, patients with chronic pain in occupational healthcare**. Three principal components explained 40% of the variation of the data, (R^2^X=0.40) with a cross-validated prediction, Q^2^X=0.17.** Figure S 5. PCA, patients with chronic pain in occupational healthcare. A: loading plot (p1/p2). B: score plot **with patients colored according to subgroup in MPI (1= adaptive coper (AC), 2= anomalous, 3= interpersonally distressed (ID), 4= hybrid, and 5= dysfunctional (Dys)). The Dys subgroup (red) was clustered mainly in the right upper quadrant in Figure B, corresponding to the loadings (items in MPI) of the upper right quadrant in the loading plot in Figure A. Dys was inversely correlated with the AC (blue) and ID subgroups in the two left quadrats in both plots.** Figure S 6. PCA, patients with chronic pain in occupational healthcare. 6A: loadings plot (p1/p3). 6B: score plot.** Patients colored according to subgroup in MPI (1= adaptive coper (AC), 2= anomalous, 3= interpersonally distressed (ID), 4= hybrid, and 5= dysfunctional (Dys)). The ID subgroup in MPI corresponded to the loadings in the lower left quadrat in the loadings plot and in the score plot.** Figure S 7A. O2PLS, patients with chronic pain in occupational healthcare**. The model comprised three predictive and one orthogonal component. The explained variance R^2^Y was 0.54 with a prediction Q^2^Y of 0.34.** Figure S 7B. O2PLS. Response variables, patients with chronic pain in occupational healthcare:** change fear (chfear), change dismissing (chdis), change secure (chsec), change physical function (chPF), change vitality (chVt), change social function (chSF), change role emotional (chRE), change mental health (chMH), change pain severity (chps), change pain interference (chpi), change life control (chlc), change affective distress (chad), change dysfunction (chdys), chBDI, chSOC, and change burnout (chpin) are illustrated according to explained variance and prediction.** Figure S 7C. O2PLS, component pq3, patients with chronic pain in occupational healthcare**. Work at follow-up (work2) correlated negatively with pain severity (ps1), pain interference (pi1), dysfunction (dys1), and support (sup1) at follow-up.** Figure S 7D. O2PLS, component pq3corr, patients with chronic pain in occupational healthcare. Correlations between loadings**. Working % at follow-up (work 2) had a correlation of 0.43 and was positively correlated with higher scores in SF-36 (SF1, SF2, PF2, PF1, Vt1, Vt2, BP1, BP2, and GH2) and negatively correlated with items in MPI (ps1, ps2, pi1, pi2, dys1, dys2, and sup1).** Table S 1A. Missing data MPI at baseline (1) and at Follow-up (2)**. Pain severity (ps), pain interference (pi), life control (lc), affective distress (ad), activity (act), dysfunction (dys), and interpersonal distress (id).** Table S 1B. Missing data at baseline (1) and at follow-up (2)**. Beck anxiety inventory (BAI), beck depression inventory (BDI), sense of coherence (SOC), Pines' burnout measure (pines), fearful attachment (fear), dismissing attachment (dism), secure attachment (sec), and preoccupied attachment (pre).** Table S 1C. Missing data at baseline (1) and at follow-up (2). Sf-36**. Physical function (PF), role physical (RP), bodily pain (BP), general health (GH), vitality (Vt), social function (SF), role emotional (RE), and mental health (MH).** Table S 2. Short Form 36 (SF-36) at baseline and at follow-up in patients with chronic pain in occupational healthcare**.* Supplemental Information about MVDA*.
