# Supplementary material for: Vulnerability and Resilience in Patients with Chronic Pain in Occupational Healthcare: A Pilot Study with a Patient-Centered Approach
Source: Pain Res Treat. 2018 Dec 2;2018:9451313. doi: 10.1155/2018/9451313 (PMC6304616; doi:10.1155/2018/9451313)
Supplement: Supplementary Materials — Supplemental Digital Content. Figure S 1. Patients in occupational healthcare. Number of patients working 100%, 75%, 50%, and 25% at baseline (blue) and at follow-up (red). At baseline 21 patients were not working versus 13 at follow-up. Figure S 2A. Patients with chronic pain in occupational healthcare. Anxiety assessed with BAI at baseline and at follow-up. BAI<16= minimal-mild anxiety, 16-25= moderate anxiety, and >25= severe anxiety. At baseline 56% of the patients had a minimal-mild anxiety versus 59.5% at follow-up. The remaining patients reported values above that level. Figure S 2B. Patients with chronic pain in occupational healthcare. Depression assessed with BDI at baseline and at follow-up. BDI<19= minimal-mild depression, 19-29= moderate depression, and >29 = severe depression. At baseline 55% of the patients had a minimal to mild depression versus 69.4% at follow-up. The remaining patients reported values above that level. Figure S 3. Patients with chronic pain in occupational healthcare. Subgroups in Multidimensional Pain Inventory (MPI) at baseline and at follow-up. Dominant subgroups were dysfunctional (Dys) and interpersonally distressed (ID). Figure S4. PCA, patients with chronic pain in occupational healthcare. Three principal components explained 40% of the variation of the data, (R2X=0.40) with a cross-validated prediction, Q2X=0.17. Figure S 5. PCA, patients with chronic pain in occupational healthcare. A: loading plot (p1/p2). B: score plot with patients colored according to subgroup in MPI (1= adaptive coper (AC), 2= anomalous, 3= interpersonally distressed (ID), 4= hybrid, and 5= dysfunctional (Dys)). The Dys subgroup (red) was clustered mainly in the right upper quadrant in Figure B, corresponding to the loadings (items in MPI) of the upper right quadrant in the loading plot in Figure A. Dys was inversely correlated with the AC (blue) and ID subgroups in the two left quadrats in both plots. Figure S 6. PCA, patients with chronic pain in oc [file 9451313.f1.docx]

**Supplementary Materials**

**Vulnerability and resilience in patients with chronic pain in occupational health care– A pilot study with a patient-centered approach**

**Figures**

**Figure S 1. Patients in occupational health care.** Number of patients working 100%, 75%, 50% and 25% at baseline (blue) and at follow-up (red). At baseline 21 patients were not working versus 13 at follow-up.

**Figure S 2A**. **Patients with chronic pain in occupational health care**. Anxiety assessed with BAI at baseline and at follow-up. BAI<16= minimal-mild anxiety, 16-25= moderate anxiety and >25= severe anxiety. At baseline 56% of the patients had a minimal-mild anxiety versus 59.5% at follow-up. The remaining patients reported values above that level.

**Figure S 2B. Patients with chronic pain in occupational health care.** Depression assessed with BDI at baseline and at follow-up. BDI<19= minimal-mild depression, 19-29= moderate depression and >29 = severe depression. At baseline 55% of the patients had a minimal to mild depression versus 69.4% at follow-up. The remaining patients reported values above that level.

**Figure S 3. Patients with chronic pain in occupational health care. Subgroups in Multidimensional Pain Inventory (MPI) at baseline and at follow-up**. Dominant subgroups were Dysfunctional (Dys) and Interpersonal Distressed (ID).

**
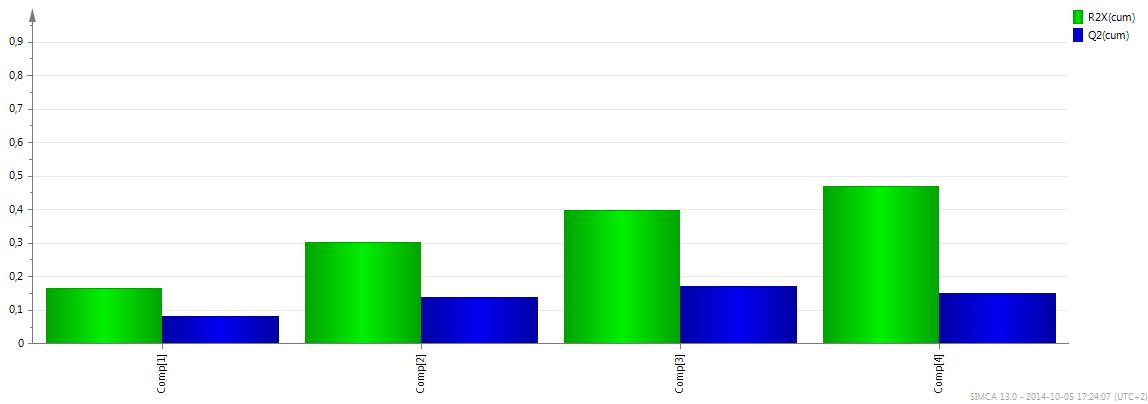
**

**Figure S 4. PCA, patients with chronic pain in occupational health care.** Three Principal components explained 40% of the variation of the data, (R^2^X=0.40) with a cross validated prediction, Q^2^X=0.17 .

 **Supplemental Figure S 5. PCA, patients with chronic pain in occupational health care. A: Loading plot (p1/p2). B; Score plot t1/t2** with patients colored according to subgroup in MPI (1= Adaptive Coper (AC), 2= Anomalous, 3= Interpersonal Distressed (ID), 4= Hybrid, 5= Dysfunctional (Dys)). The Dys subgroup (red) was clustered mainly in the right upper quadrant in Figure B, corresponding to the loadings (items in MPI) of the upper right quadrant in the loading plot in Figure A. The Dys subgroup was inversely correlated with the AC (blue) and ID (green) subgroups in the two left quadrats in both plots.

**Figure S 6. PCA, patients with chronic pain in occupational health care. S6A. Loading plot (p1/p3). S6B: Score plot t1/t3.** Patients colored according to subgroup in MPI (1= Adaptive Coper (AC), 2= Anomalous, 3= Interpersonal Distressed (ID), 4= Hybrid, 5= Dysfunctional (Dys)).The ID subgroup in MPI corresponded to the loadings in the lower left quadrat in the loading plot and the in the corresponding score plot, inversely correlated with a Dys subgroup in the upper right quadrants in the score plot


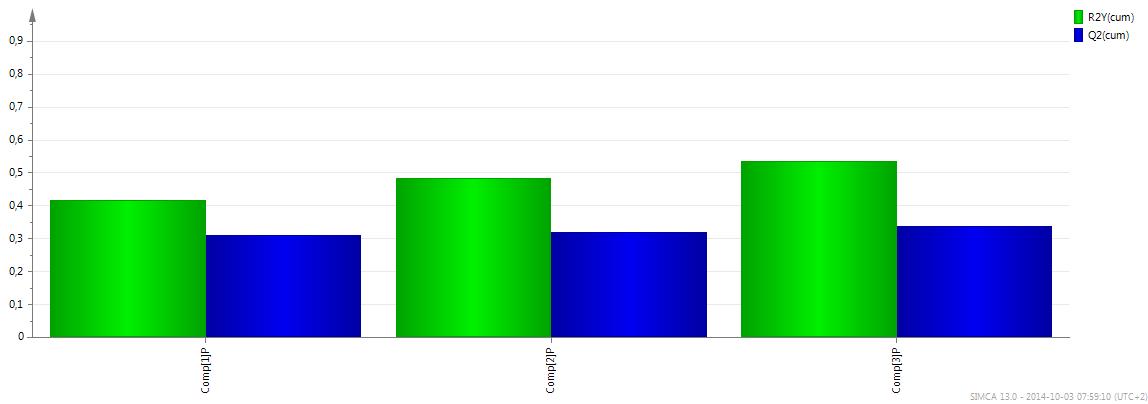


**Figure S 7A.** **O2PLS, patients with chronic pain in occupational health care**. The model comprised three predictive and one orthogonal component. The explained variance R^2^Y was 0.54 with a prediction Q^2^ Y of 0.34


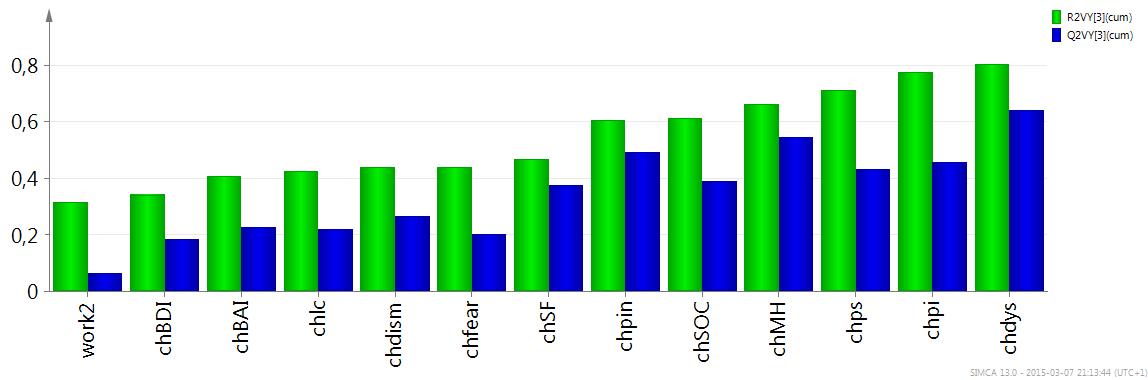


**Figure S 7B. O2PLS response variables, patients with chronic pain in occupational health care:** change fear (chfear), change dismissing (chdis), change secure (chsec), change Physical function (chPF), change Vitality(chVt), change Social function (chSF), change Role emotional (chRE), change Mental health (chMH), change Pain severity (chps), change Pain interference (chpi), change Life control (chlc), change Affective distress (chad), change Dysfunction (chdys), chBDI, chSOC, Change burnout (chpin), are illustrated according to explained variance and prediction.

**Figure S 7C. O2PLS, Component 3, patients with chronic pain in occupational health care.** Work at follow-up (work2) correlated negatively with pain severity (ps1), pain interference (pi1), dysfunction (dys1) and support (sup1) at follow-up.

**Figure S 7D. O2PLS, Component 3, patients with chronic pain in occupational health care. Correlations between loadings.** Working % at follow-up (work 2) had a correlation of 0.43 and was positively correlated with higher scores in SF-36 (SF1, SF2, PF2,PF1, Vt1,Vt2, BP, BP2, GH2) and negatively correlated with items in MPI (ps1, ps2, pi1, pi2, dys1, dys2, sup1).

**Tables**

**Table S 1A. Missing data MPI at baseline (1) and at follow-up (2).** Pain severity (ps). Pain interference (pi), Life control (lc), Affective distress (ad), Activity (act), Dysfunction (dys), Interpersonal distress (id)

| Patients  (n) | ps1 | ps2 | pi1 | pi2 | lc1 | lc2 | ad1 | ad2 | act1 | act2 | dys1 | dys2 | Id1 | Id2 |
| --- | --- | --- | --- | --- | --- | --- | --- | --- | --- | --- | --- | --- | --- | --- |
|  | 42 | 38 | 42 | 38 | 41 | 38 | 42 | 38 | 42 | 38 | 42 | 38 | 39 | 35 |
|  |  |  |  |  |  |  |  |  |  |  |  |  |  |  |

**Table S 1B**, **Missing data at baseline (1) and at follow-up (2)**. Beck anxiety inventory (BAI), Beck depression inventory (BDI), Sense of coherence (SOC), Pines’ burnout mesasure (pines), fearful attachment (fear), dismissing attachment (dism), secure attachment (sec), preoccupied attachment (pre)

| patients | BAI  1 | BAI  2 | BDI  1 | BDI  2 | SOC  1 | SOC  2 | Pines  1 | Pines  2 | Fear  1 | Fear  2 | Dism  1 | Dism2 | Sec1 | Sec2 | Pre1 | Pre  2 |  | Pre1 | Pre2 |
| --- | --- | --- | --- | --- | --- | --- | --- | --- | --- | --- | --- | --- | --- | --- | --- | --- | --- | --- | --- |
|  | 41 | 37 | 40 | 36 | 40 | 38 | 39 | 38 | 39 | 37 | 39 | 37 | 39 | 37 | 39 | 37 |  |  |  |

**Table S 1C**, **Missing data at baseline (1) and at follow-up (2)**. SF-36

| Patients  (n) | PF1 | PF2 | RP1 | RP2 | BP1 | BP2 | GH1 | | GH2 | Vt1 | Vt2 | SF1 | SF2 | RE1 | RE2 | MH1 | MH2 |
| --- | --- | --- | --- | --- | --- | --- | --- | --- | --- | --- | --- | --- | --- | --- | --- | --- | --- |
|  | 42 | 35 | 40 | 37 | 41 | 36 | | 40 | 35 | 40 | 36 | 40 | 36 | 41 | 35 | 40 | 36 |

**Table S 2.** Short form 36 (SF-36) at baseline and at follow-up in patients with chronic pain in occupational health care.

|  | Baseline (n=42)  Mean (SD)  Range | Follow-up (n=35-36)  Mean (SD)  Range | *p* value |
| --- | --- | --- | --- |
| Physical Function | 52.1 (20.74)  5-87.5 | 55.6 (22.09)  5-90 | 0.22 |
| Role Physical | 12.5 (25.94)  0-100 | 13.6 (22.96)  0-100 | 0.67 |
| Bodily Pain | 23.3 (14.22)  0-52 | 29.8 (15.87)  0-74 | 0.11 |
| General Health | 38.2 (18.00)  10-82 | 41.0 (19.10)  5-87 | 0.25 |
| Vitality | 23.8 (18.30)  0-73.3 | 31,8 (19.50)  0-75 | 0.07 |
| Social Function | 53.4 (27.15)  0-100 | 56.9 (27.14)  25-100 | 0.42 |
| Role Emotional | 63.4 (42.04)  0-100 | 63.81(41.52)  0-100 | 0.92 |
| Mental Health | 57.1(24.19)  0-100 | 63.2 (22.10)  8-100 | 0.06 |

**Suplemental Information about MVDA**

A Principal Component Analysis (PCA) [[1](#_ENREF_1)]was performed using the SIMCA-P + v13.0 software (Umetrics Sweden).The data matrix included all patients as objects using the estimated data for all variables as loadings. Before calculation of principal PCA components, all data were mean centered and unit variance scaled. The number of significant components were estimated by cross-validation, i.e. by Jack-knifing [[2](#_ENREF_2)], where parts of data, i.e. one seventh, are omitted such that model variability can be predicted. Furthermore there was an inspection of the pattern of Eigenvectors, which level out when most structured data are included in the system. Model scores, i.e. patients, and tested variables, i.e. loadings, were visualized in a multivariate coordinate system and the multivariate projection was made by means of factor projections to new orthogonal coordinate axes such that a number of principal components were retrieved from the data. The combination of loadings and scores were thereafter used to investigate obtained patterns from patient groups and for clinical interpretation.

Orthogonal Partial Least Squares (OPLS and O2PLS) are multivariate regression techniques for modeling the optimal correlation between the factors or predictors (X) and responses (Y) with similarities to PCA [[3](#_ENREF_3),[4](#_ENREF_4)], The Two-way Orthogonal Partial Least Squares (O2PLS) is bidirectional. Therefore X can be used to interpret Y and Y can be used to interpret X. The systematic variability in O2PLS can be divided into four parts: The X/Y joint variation, the Y-orthogonal variation in X, the X-unrelated variation in Y and noise [[4](#_ENREF_4)] . Before OPLS and O2PLS scores and loadings, i.e. t and p vectors, were generated, the X-matrix is biased by the response related weight vectors w*, estimating the correlation structure between X and Y expressed in the same format as the model loadings p. Subsequently the w*-vector was used to generate the first scores (t_1_) and the loadings (p_1_) [[3](#_ENREF_3)] . OPLS and O2PLS model complexity was estimated by factor validity using cross-validation [[2](#_ENREF_2)]. For all OPLS and O2PLS models, data are invariably subjected to mean centering and unit variance scaling of the data before model generation.

**References**

1. Jackson JE (1991) A user's guide to principal components. New York: Willey.

2. Wold S (1978) Crossvalidation-estimations of the number of components in factor and principal components models. Technometrics 20: 397-405.

3. Trygg J, Wold S (2002) Orthogonal Projections to Latent Structures (OPLS). Journal of Chemometrics 16: 119-128.

4. Trygg J, Wold S (2003) O2-PLS for Qulitative and Quantitative Analys in Multivariate Calibration. Journal of Chemometrics 16: 283-293.
